# Dental Treatment Needs of Permanent First Molars in Mashhad Schoolchildren

**DOI:** 10.5681/joddd.2010.014

**Published:** 2010-06-24

**Authors:** Masoumeh Ebrahimi, Behjat-Al-Molook Ajami, Ali Reza Sarraf Shirazi, Monavar Afzal Aghaee, Somayeh Rashidi

**Affiliations:** ^1^ Assistant Professor, Department of Pediatric Dentistry, Faculty of Dentistry, Mashhad University of Medical Sciences, Iran; ^2^ Associate Professor, Department of Pediatric Dentistry, Faculty of Dentistry, Mashhad University of Medical Sciences, Iran; ^3^ Statistics Consultant, Mashhad University of Medical Sciences, Iran; ^4^ Dentist, Private Practice

**Keywords:** Caries prevalence, first molar, schoolchildren, dental treatment needs

## Abstract

**Background and aims:**

In spite of their enormous importance, permanent first molars might be affected by caries in children in developing countries. The aim of this study was to evaluate the treatment needs of first permanent molars in a group of schoolchildren in Mashhad.

**Materials and methods:**

This cross-sectional descriptive study was carried out on 700, 7-9 year-old students in primary schools in Mashhad. The schools were randomly selected from each district. Treatment needs and DMFT of first perma-nent molars were calculated. Data was analyzed using ANOVA, Chi-Square and t-test.

**Results:**

A total of 95.3% of the children required dental treatment. Fissure sealant application and filling were the treat-ments most required in all age groups. The mean DMFT of first permanent molars was 1.31±1.4. It was significantly higher in girls than boys (P=0.040).

**Conclusion:**

Great treatment needs and caries prevalence in permanent first molars in Mashhad schoolchildren show that dental caries is still a serious problem in the children of our society; therefore, education of parents and teachers is necessary for promoting children's oral health.

## Introduction

From a functional and developmental point of view, the first permanent molars are the most important teeth, with a key role in occlusion. The role of first permanent molars is established in balanced occlusion.^1^ Loss of first permanent molars, because of dental caries, negatively affects both arches and has adverse effects on occlusion. It is reported that early extraction of these teeth results in tilting of neighboring teeth to hollow spaces, supereruption of the teeth in the opposite arch, unilateral chewing, shift in midline and dental malocclusion.^2^

Premature contact, horizontal mandibular displacement and continuous displacement of the condyles during growth and development following loss of mandibular first permanent molar lead to asymmetric growth of the mandible. In addition, posterior cross-bite following extraction of maxillary first permanent molars might induce mesialization and rotation of posterior teeth in growing children. Gaglaroglu reported skeletal and dental asymmetries following early extraction of a first permanent molar.^1^ Furthermore, early loss of first permanent molars creates periodontal problems.^3^ Based on these studies permanent first molars are of utmost importance in children.^1 - 3^ On the other hand, although the prevalence of dental caries among children has decreased in the developed world, there has been an increase in the prevalence of caries in some developing countries.^4^ The majority of low-income families do not have access to sufficient resources to provide essential health care for their children.^5^ Itshould be noted that occlusal surfaces of permanent first molars has several pits and fissures that make them susceptible to dental caries. Besides, it is reported that occlusal caries comprise 90% of dental caries in children and adolescents.^6^ In addition, they are the first permanent teeth which erupt in the posterior area of the oral cavity.^7^ Because of paramount importance of permanent first molars and to prevent the above-mentioned complications following early loss of these teeth, we decided to evaluate the treatment needs of permanent first molars among 7-9 year-old students in Mashhad primary schools.

## Materials and methods

This cross-sectional descriptive study was carried out on 700, 7-9 year-old students from 14 male and female primary schools, in Mashhad in 2007. A simple random sampling method was used to select the study group. The Ethics Committee of Mashhad University of Medical Sciences approved this research (#85437). Approval from the authorities was obtained before the study. The parents were sent an information sheet about the study, and only children with informed consent forms were evaluated. The World Health Organization (WHO) oral health form was used to record data.^8^ Dental examination was carried out by one senior dental student from Mashhad Faculty of Dentistry. Prior to the study, the examiner took part in a training and clinical calibration exercise with principal investigators. Twenty children were examined twice for reliability regarding the presence of caries on each tooth. The agreements between the first and second examinations were found to be good. Oral examination was performed with a mouth mirror, an explorer and a halogen lamp in suitable places in schools. No radiographs were taken; DMFT and treatment needs for permanent first molars were calculated. Data was analyzed with ANOVA, chi-square and t-test.

## Results

A total of 700, 7-9 year-old primary school students were surveyed, consisting of 350 (50%) boys and 350 (50%) girls. All the subjects were in the mixed dentition period. The mean DMFT of the permanent first molars was 1.31±1.4. The t-test was used to compare DMFT between genders. It was significantly higher in girls (DMFT=1.41±1.5) than boys (DMFT=1.2±1.3) (P=0.040). ANOVA was used to compare DMFT between the three age groups. DMFT increased significantly with age (P=0.001). The average of DMFT for permanent first molars and its components among the schoolchildren are summarized based on age in Table 1. Table 2 shows the results for dental treatment needs by age and gender. A total of 95.3% of the study population required some type of treatment. From 700 children examined, 590 (84.3%) needed fissure sealant, and 372 (53.1%) required fillings. The need for pulp care (2.4%), crown placement (1.7%), extraction (0.4%) and orthodontic treatment (4.7%) was minimal. Chi-square was used to evaluate the relationship between sex and type of treatment needs. There were significant differences in needs for fissure sealant, fillings and orthodontic treatment in different ages (P<0.05). In addition, boys needed crown placement and orthodontic treatment more than girls (P<0.05) (Table 3).

**Table 1:** DMFT (mean ± SD) in permanent first molar and its components, by age

Indices	7 years-old	8 years-old	9 years-old	Total
D	0.69±1.04	1.23±1.43	1.7±1.40	1.19±1.38
M	0.00±0.00	0.00±0.00	0.00±0.00	0.00±0.00
F	0.07±0.44	0.09±0.51	0.18±0.68	0.11±0.55
DMFT	0.76±1.4	1.32±1.47	1.87±1.4	1.31±1.4

**Table 2:** Dental treatment needs in permanent first molars, by age and gender

Age & gender	Fissure sealant	Filling	Pulp care	Crown placement	Extraction	Orthodontic
**7 years** Boys Girls Total	89.2 78.8 84.0	32.5 34.7 33.6	2.5 2.5 2.5	2.5 0.0 1.3	0.0 0.0 0.0	5.8 0.8 3.4
**8 years** Boys Girls Total	90.5 86.4 88.4	51.7 58.4 55.2	3.4 0.8 2.1	2.6 0.8 1.7	0.9 0.0 0.4	5.2 1.6 3.3
**9 years** Boys Girls Total	81 79 80.1	74.1 69.5 71.9	4.3 1 2.7	3.4 1 2.3	1.7 0.0 0.9	9.5 5.7 7.7

**Table 3:** Dental treatment needs in first permanent molars, by gender

Gender	Fissure sealant	Filling	Pulp care	Crown placement	Extraction	Orthodontic
Girls Boys Total	81.6 86.9 84.3	53.7 52.6 53.1	1.4 3.4 2.4	0.6 2.8* 1.7	0.00 0.9 0.4	2.6 6.8* 4.7

*=P<0.05

## Discussion

A total of 700 students (350 boys and 350 girls) with an age range of 7-9 years were surveyed from 14 primary schools in Mashhad. The mean DMFT in permanent first molars was 1.31±1.4 in 7-9 year-old children in the present study. A limited number of studies can be found in literature review on treatment needs in permanent first molars; therefore, we discuss nearly-related studies. Mean DMFT in 6-year-old children in Spain was 0.25±0.71 and 1.50±1.67 in 9-year-old children.^9^ In addition, mean DMFT in permanent dentition in 7-8 year-old children was 0.35± 0.77 and 0.68±0.99 in 9-10 year-old children in India.^10^ Besides, Naidu reported that a DMFT of 2.54±3.12 in 6-8 year-olds in Trinidad and Tobago.^11^DMFT in the present study was more than that in Saravanan and Varez-Arenal studies and less than that in Naidu study. It should be pointed out that the only permanent teeth in 7-9 year-old children are the first molars and anterior teeth;^7^however, presence of dental caries in anterior teeth at this age range is rare. Therefore, the results of these studies are nearly comparable to those of the present study. Another difference between Saravanan’s study and the present study is that their study group consisted of rural children. There was an increase in mean DMFT in permanent first molars with age. The mean DMFT score was higher (1.41±1.5) in girls than in boys (1.2±1.3), in accordance with other studies.^10 , 12^ Differences between the sexes might be attributed to the earlier eruption of these teeth in girls than in boys.^13^

Our results showed that 95.3% of children needed some type of treatment, which is more than other studies.^9 - 11 , 14^ There were significant differences in needs for fissure sealant, fillings and orthodontic treatment between different age groups (P<0.05). In our study, 8-year-old children had the greatest need for fissure sealant (P=0.040), and 9-year-old children had the greatest need for fillings (P=0.000) and orthodontic treatment (P=0.040).

It was found that boys had more need for fissure sealant, pulp care, extraction and orthodontic treatment in permanent first molars, but needs for crown placement and orthodontic treatment were significant (P<0.05). As a result, boys had more treatment needs than girls, which might be attributed to a greater attention paid to oral health by girls in comparison with boys. There was less need for dental fillings in permanent first molars in boys than in girls, but the difference was not significant.

It was concluded that 77.6% of 6-year-olds and 70.2% of 9-year-olds in Spain needed dental fillings. Furthermore, 7.7% of 6-year-olds and 11% of 9-year-olds needed pulp care. The need for extraction was 8.3% and 13.5% in 6- and 9-year-olds, respectively.^9^ Treatment needs for primary and permanent teeth have been evaluated in a number of studies and our study has only evaluated treatment needs in permanent first molars. Nalweyiso et al. concluded that 52.5% of 5-7 year-olds in Uganda needed fillings and almost one-third needed tooth extractions,^14^with differences in age range with our study; we also evaluated treatment needs in first permanent molars.

Saravanan et al. reported that 25.3% of 5-10 year-old schoolchildren with permanent dentition in South India need one-surface fillings, 1.6% need two or more surface fillings and 0.2% were in need of extractions; the need for fissure sealant, crown placement, and pulp care was 12.6%, 1.2%, and 0.2%, respectively. There was a strong need for one-surface restorations.^10^ Furthermore, Naidu et al. showed that 72% of 6-8 year-olds in Trinidad and Tobago had some treatment needs (29% one-surface fillings, 46% two or more surface fillings, 29% fissure sealant, 18% extractions, 1% crowns).^11^ It should be pointed out that they evaluated treatment needs in primary and permanent dentitions. The high level of caries experience and the need for fillings in permanent first molars in the present study shows a low level of parental awareness about the importance of permanent first molars and the oral health of children. Parents and teachers must be instructed in the importance of permanent first molars in the oral cavity, oral hygiene, decreasing the frequency of sugar consumption, use of multiple delivery systems of fluoride and routine checkups. It should be emphasized that insurance policies do not cover the costs of dental treatments and preventive measures which play an important role in the lack of access to oral health service systems in our society.

## Conclusions

High treatment needs and caries prevalence in permanent first molars in Mashhad schoolchildren show that dental caries is still an important problem in the children of our society; therefore, educating the parents and teachers on the importance of permanent first molars and promoting children's oral health care is necessary.
